# Can We Simplify Liposome Manufacturing Using a Complex DoE Approach?

**DOI:** 10.3390/pharmaceutics16091159

**Published:** 2024-09-01

**Authors:** Sarah Lindsay, Olympia Tumolva, Tatsiana Khamiakova, Hans Coppenolle, Martin Kovarik, Sanket Shah, René Holm, Yvonne Perrie

**Affiliations:** 1 Strathclyde Institute of Pharmacy and Biomedical Sciences, University of Strathclyde, 161 Cathedral Street, Glasgow G4 0RE, UK; sarah.lindsay@strath.ac.uk; 2 Global Development, Janssen Pharmaceutica NV, a Johnson & Johnson Company, Turnhoutseweg 30, 2340 Beerse, Belgium; 3 Therapeutics Development and Supply, Janssen Pharmaceutica NV, a Johnson & Johnson Company, Turnhoutseweg 30, 2340 Beerse, Belgium; 4 Department of Physics, Chemistry, and Pharmacy, University of Southern Denmark, Campusvej 55, 5230 Odense, Denmark

**Keywords:** liposomes, microfluidics, design of experiments, manufacturing, machine learning (ML)

## Abstract

Microfluidic liposome production presents a streamlined pathway for expediting the translation of liposomal formulations from the laboratory setting to clinical applications. Using this production method, resultant liposome characteristics can be tuned through the control of both the formulation parameters (including the lipids and solvents used) and production parameters (including the production speed and mixing ratio). Therefore, the aim of this study was to investigate the relationship between not only total flow rate (TFR), the fraction of the aqueous flow rate over the organic flow rate (flow rate ratio (FRR)), and the lipid concentration, but also the solvent selection, aqueous buffer, and production temperature. To achieve this, we used temperature, applying a design of experiment (DoE) combined with machine learning. This study demonstrated that liposome size and polydispersity were influenced by manipulation of not only the total flow rate and flow rate ratio but also through the lipids, lipid concentration, and solvent selection, such that liposome attributes can be in-process controlled, and all factors should be considered within a manufacturing process as impacting on liposome critical quality attributes.

## 1. Introduction

Liposomal drug delivery systems have the potential to improve treatments of a variety of diseases through targeted delivery. Since their discovery in 1961, liposomes have become the most extensively researched drug delivery system owing to their unique ability to transport a large variety of drugs [1]. Despite their proven ability to improve the delivery and efficacy of drugs, the clinical application of liposomes remains limited due to the cost and complexity of their manufacturing process. Conventional manufacturing methods use a top-down approach, which involves the breakdown of initial material to the desired size through a series of processing steps. In order to obtain a uniform and monodisperse population, further downstream processing is required, which is unsuitable for many active pharmaceutical ingredients due to the high-shear forces involved in the manufacturing process [2]. Therefore, new manufacturing techniques that are less time-consuming and scale-independent, which might precisely and easily control manufacturing of liposomal products, are being investigated.

Among the different manufacturing methods that have been investigated for liposome manufacturing, microfluidics offers many advantages, including low cost and flexibility. Microfluidic devices promote self-assembly of the liposomes through rapid mixing of the organic solvent and aqueous phases, and hydrodynamic manipulation of the fluids through chaotic advection [3,4]. In contrast to conventional methods, microfluidics uses a bottom-up approach, which addresses some of the limitations associated with the top-down approaches, such as high polydispersity, batch-based production, and user variability [2]. Microfluidic mixers can be made from various materials, such as polymers, silicon, or glass, and can be manufactured in various ‘designs’ [4]. There are two types of microfluidic mixing: passive and active mixing. Passive mixing relies on the channel geometry of the mixer to increase the contact area and time for enhanced diffusion of the aqueous and organic phases, whereas active mixers incorporate external forces within the microfluidic device to increase the mixing efficiency [5]. The fluid flow within these mixers can then also be classified as either turbulent or laminar and, in order to understand and characterize the fluid flow within these mixers, there are a couple of parameters that need to be determined, including microflows and dimensionless numbers. There are two dimensionless numbers that are often used to describe microfluidic systems, the Peclet number and Reynolds number [6]. The Peclet number is a measure of the molecular diffusion between convective and advective diffusion processes and is critical for optimization of passive micromixers [6]. The diffusion of the two fluid streams occurs at the interface of where these two liquids meet and can often be a slow process, requiring an increased channel length for sufficient mixing. The larger the Peclet number, the shorter the length required for efficient mixing. The Reynolds number is defined as the ratio between inertial forces and viscous forces and is an indicator of the type of flow within the microfluidic channels. A Reynolds number of <2000 is considered to be laminar flow and >2000 is considered to be turbulent flow [7].

In general, an organic solvent stream containing the lipids is forced through a central channel, where it is then intersected on either side by the aqueous buffer stream. This hydrodynamic flow focusing (HFF) method was first described by Jahn et al. in 2004 [8] and has since been modified to produce the staggered herringbone micromixer (SHM) [9]. Similar to HFF, the SHM involves the controlled mixing of an organic solvent with an aqueous buffer; however, the design of this micromixer uses rapid mixing through chaotic advection induced by injecting the solvent and aqueous streams into a Y-shaped mixer, in which the two fluid streams are passed over a series of protruding herringbone structures within the channel [4]. The most notable feature of this microfluidic technique is the ability to control the aqueous:solvent mixing ratio, also known as the flow rate ratio (FRR), as well as the speed at which these two fluid streams mix, known as total flow rate (TFR). The formation of liposomes by microfluidics is driven by the diffusion of the organic solvent in the aqueous buffer and, therefore, liposome characteristics, such as particle size, can be controlled through the manipulation of the FRR and TFR.

Previous studies have investigated the importance of both TFR and FRR, as well as various formulation parameters, such as the lipid and solvent, on the resultant liposome properties, e.g., polydispersity and particle size. There have been numerous research papers demonstrating the importance of FRR on the liposome particle size, with increasing FRR resulting in smaller liposomes [10,11,12]. TFR has been reported to have minimal impact on resultant liposome characteristics, demonstrating that microfluidics is capable of high-throughput production with minimal impact on the resultant liposomes [12,13,14]. Solvent selection has also been shown to have an important impact on the resultant liposome size and PDI [15]. Research carried out by Webb et al. has shown that increasing the solvent polarity results in smaller particle sizes [15]. This was suggested to be due to the rate of polarity change, with an increased rate of change in the polarity during microfluidic mixing causing smaller lipid disc formation and, therefore, smaller liposomes [15]. Recent design of experiment (DoE) studies has been carried out investigating the relationship between FRR, TFR, as well as solvent and lipid composition [13,16]; however, these studies have used limited FRRs, TFRs, and lipid concentrations. Therefore, in order to further understand the important parameters for microfluidics in the liposome manufacturing, the aim of the present study was to investigate the use of microfluidic production for liposomes and identify the relationship between not only TFR, FRR, and lipid concentration, but also solvent selection, aqueous buffer, and production temperature, applying a design of experiment (DoE) combined with machine learning, thereby mapping as many formulation and process parameters as possible within one study, allowing a deep and unique cross-factorial analysis.

## 2. Materials and Methods 

### 2.1. Materials

The 1,2-dioctadecanoyl-*sn*-glycero-3-phosphocholine (DSPC), 1,2-dioleoyl-sn-glycero-3-phosphocholine (DOPC), and cholesterol (chol, >99%) were obtained from Sigma-Aldrich Ltd. (Poole, UK). The 1,2-distearoyl-sn-glycero-3-phosphoethanolamine-N-[methoxy(polyethylene glycol)-2000] (sodium salt) (DSPE-PEG2000, >99%) was obtained from Lipoid (Ludwigshafen, Germany). Ammonium sulfate ([NH_4_]_2_SO_4_, >99%) was obtained from Sigma-Aldrich Ltd. (Poole, UK). Phosphate-buffered saline tablets (10 mM, PBS pH 7.3) were acquired from Oxoid Ltd. (Basingstoke, UK). All reagents (ethanol, methanol, and isopropyl alcohol) were of analytical grade (HPLC grade, >99.8%) and Transcutol was of general-purpose grade and purchased from commercially available suppliers. Dialysis tubing with a molecular weight cut-off of 12–14 kDa was purchased from Sigma-Aldrich Ltd. (Poole, UK).

### 2.2. Microfluidic Production of Liposomes

#### Preparation of Liposomes Using a Bench-Scale System

PEGylated liposomes were prepared using the bench-scale microfluidic system (NanoAssemblr Benchtop) from Precision NanoSystems Inc. (Vancouver, ON, Canada) using a staggered herringbone micromixer (SHM). Individual lipid stocks were prepared at concentrations of 2 to 40 mg/mL in either ethanol, methanol, 2-propanol (IPA), or Transcutol, and contained either DOPC or DSPC, cholesterol, and DSPE-PEG2000 at a w/w ratio of 3:1:1, respectively. Lipids were heated for solubilization but were stable once in solution. The lipids were heated to either 20 °C or 60 °C before injection into the micromixer, according to the experimental plan. Ammonium sulfate ([NH_4_]_2_SO_4_, 250 mM) or phosphate-buffered saline (10 mM) was used as the aqueous phase. Liposomes were produced at a total flow rate (TFR) of 5, 12.5, or 18 mL/min and a flow rate ratio (FRR) of 1:1, 10:1, or 19:1 (aqueous:organic). Formulations were dialyzed (MWCO 12,000–14,000 Da, Sigma-Aldrich, Poole, UK) for 1 h under magnetic stirring against 200 mL of PBS or ammonium sulfate buffer.

### 2.3. Characterization of Particle Size and Polydispersity Using Dynamic Light Scattering

The particle sizes, measured as the hydrodynamic diameters, polydispersity indexes (PDI), and zeta potentials were measured by dynamic light scattering using a Zetasizer Nano ZS (Malvern Instruments Ltd., Worcestershire, UK) equipped with a 633 nm laser and a detection angle of 173°. The samples were measured at 25 °C in the buffer mapped to each formulation and the values of either PBS or ammonium sulfate were used for refractive indexes (1.335 and 1.52, respectively) and viscosity (1.02 cP and 1.338 cP, respectively). Zetasizer Software v.7.11 (Malvern Instruments Ltd., Malvern, Worcestershire, UK) was used for acquisition of data.

### 2.4. Statistical Methodology

#### 2.4.1. DoE

The study design involved setting up an I-Optimal DoE [17] with 135 experimental runs, including control runs to check the reproducibility of the process and measurement. The DoE has a possibility to estimate several third-order and fourth-order interaction terms. Additionally, 18 validation runs were created to check how well the statistical model predicted the PSD and PDI on new combinations of factor settings that were not used in the first DoE.

#### 2.4.2. Statistical Modeling

As the current dataset was obtained via I-Optimal DoE, the multiple linear regression models as well as regularized linear regression models (such as Elastic Net and Lasso) were considered. The primary linear regression model had very good performance on the training set and high error on the validation set of experiments, the phenomenon known in statistical data analysis as overfitting. To overcome the overfitting, regularized linear models were employed. Application of regularized models allows to avoid overfitting and perform model selection at the same time, as the least relevant model terms would be set to zero. The resulting prediction error of regularized regression using Elastic Net was higher compared to the original multiple linear regression model; however, the prediction error was comparable for training and validation sets, which justified its use from the prediction perspective. Additional modeling efforts included machine learning methods, such as Random Forests and XGBoost, but their performance in terms of prediction error was not better compared to the Elastic Net models. Full technical details on the modeling are provided in the Supplementary Materials.

#### 2.4.3. Visualization of Modeling Results

Due to complexity of DoE and the final selected models involving higher-order interactions, which were relevant for prediction, contour plots were constructed for ease of interpretation. A typical contour plot would display the average response surface in terms of continuous factors, TFR and FRR, for different combinations of categorical factors: solvent, buffer, lipid, and discretized lipid concentrations, and temperatures. Contour plots allow to visualize and put emphasis on the complex interactions; however, the model uncertainty (i.e., average prediction error) was not considered. 

In addition, the measured data points from training and validation sets were added to the contour plots to demonstrate how the final selected model fit measurements from both sets of results.

## 3. Results

To investigate the individual effects of, and the interactions between, formulation and process parameters, a DoE was set up with a total of 135 runs (plus an additional 9 control runs carried out weekly). The testing parameters are shown in Table 1, and the DoE analysis highlighted the simulated key factors based on this workspace. The TFR and FRR ranges that the NanoAssemblr Benchtop is capable of running are at a TFR of 1–20 mL/min and a FRR from 1:1 to 20:1, and a TFR of 18 mL/min and FRR of 19:1 were set as the maximum due to constraints with the syringe sizes available. The concentration range was selected based on the lipid solubility in the chosen solvents. The exact concentration of each lipid in the solvents at both 20 °C and 60 °C can be found in the Supplementary Materials (Supplementary Table S3).

### 3.1. Exploration of Overall Effects of DoE Factors

**Figure 1:**
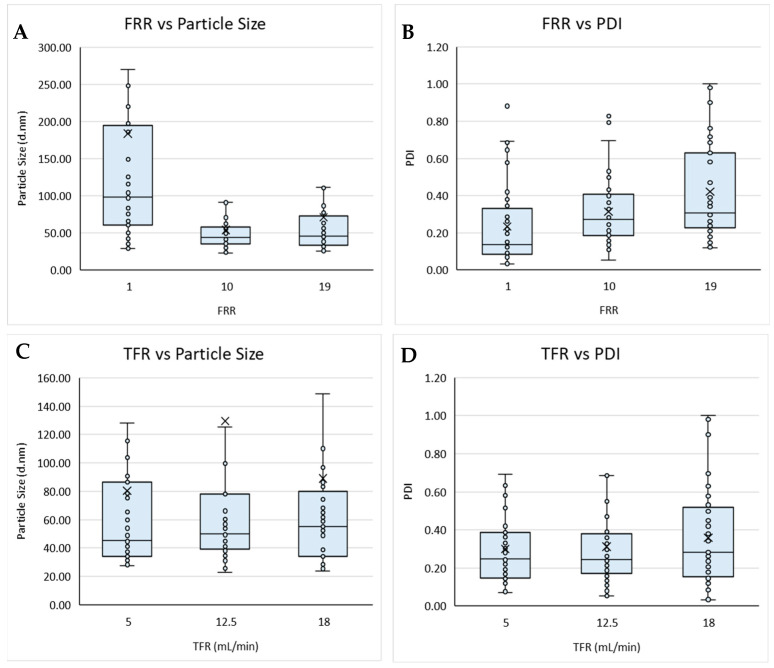
Boxplots of the flow rate ratio (FRR) against (**A**) particle size and (**B**) PDI, and total flow rate (TFR) against (**C**) particle size and (**D**) PDI for liposomes prepared via microfluidics following a DoE. Each point represents a result from an individual run. The outlier points have been removed to obtain a clearer view of the mean effect of each parameter. Factors tested are shown in Table 1.

**Figure 2:**
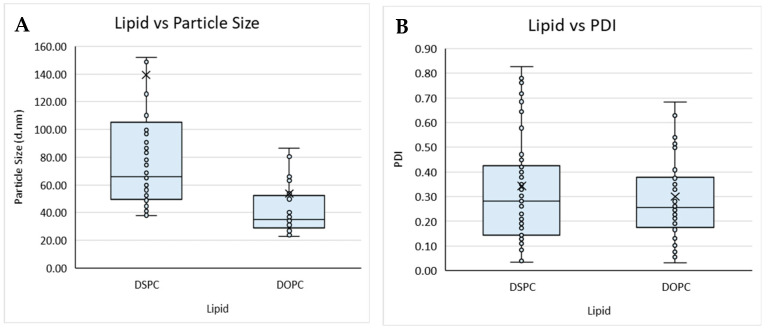

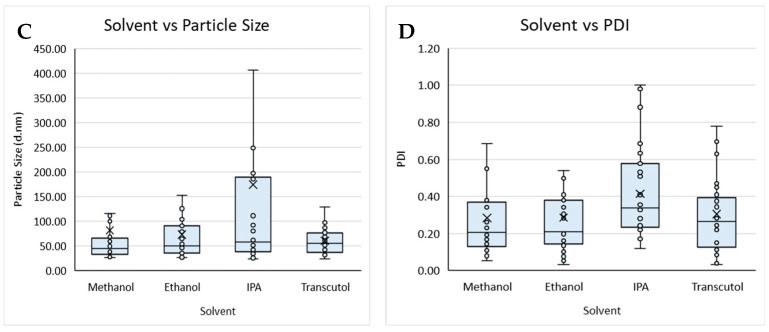
Boxplot of lipid composition against (**A**) particle size and (**B**) PDI, and solvent against (**C**) particle size and (**D**) PDI for liposomes prepared by microfluidics following the DoE. Each point represents a result from an individual run. The outlier points have been removed to obtain a clearer view of the mean effect of each parameter. Factors tested are shown in Table 1.

To highlight the overall (marginal) effects of the investigated process and formulation parameters on PDI and PSD, the boxplots were constructed (Figure 1 and Figure 2). These boxplots show the distribution of particle size or PDI for different levels of a given parameter without considering the levels of other parameters in the study. It must be noted that no clear difference in response values on the boxplot does not exclude a situation when a parameter in question had a meaningful impact in combination with specific settings of other parameters.

#### 3.1.1. Process Variables

Initially, the process variables of total flow rate, flow rate ratio, and production temperature were considered (Figure 1). Based on the boxplots, the TFR and production temperature showed no obvious overall effects on either the size nor PDI of the resultant liposomes produced; however, the FRR appeared to affect both the size and PDI (Figure 1A,B). These results also showed a wider data distribution at FRR 1:1 for particle size, with FRR 1:1 resulting in an overall larger particle size (Figure 1A), whilst the highest FRR yielded the widest spread for PDI (Figure 1B). These results aligned with previous research, showing that FRR had a greater influence over particle size, when compared with TFR (Figure 1C,D), and that increasing the FRR resulted in smaller particles but high dilutions can result in more heterogenicity [13,16,18]. The TFR is responsible for the speed at which the two fluid streams meet and are mixed. At a high TFR, the mixing efficiency may be reduced due to a shorter overall duration of mixing, resulting in less uniform particles being produced. It is also important to note that Figure 1C,D does not take into account the FRR at which the particles were produced. Therefore, the slight increase in PDI observed in Figure 1D could also be attributed to more particles being produced at low FRR, which can also result in an increase in PDI.

#### 3.1.2. Formulation Variables

The lipid composition of liposomes and the buffers used within the formulation can determine the loading efficiency, targeting, and circulation time of the resultant product and, therefore, needs to be carefully considered from a manufacturing perspective. Therefore, to study this, we tested different lipid compositions (DSPC vs. DOPC; Table 1) with different solvents (Transcutol, methanol, ethanol, and IPA) and considered their size and PDI (Figure 2).

Overall, the buffer did not impact on size nor PDI, whereas the lipid used impacted particle size, with liposomes composed of DSPC (Figure 2A) showing an overall larger particle size when compared to liposomes produced with DOPC (Figure 2A,B). The organic solvent used also impacted the particle size and PDI of the liposomes produced from a Transcutol lipid stock, having an overall smaller mean size when compared to the liposomes produced with IPA, which produced the largest mean size. Liposomes produced from methanol, ethanol, and Transcutol all had similar average PDIs; however, those produced from IPA had an overall larger average PDI. Lopez and coworkers [19] reported that liposomes produced from a Transcutol stock were smaller than those produced from an ethanol stock independent of the production temperature and lipid concentration, in contrast to the data reported in this work.

When investigating differences in the obtained particle size and PDI as a function of lipid concentration, it was difficult to determine whether there was any effect or not, as the concentration of the lipid stock used depended on the lipid solubility in each solvent and the production temperature. Therefore, to visualize the effect of lipid concentration on liposomes produced by microfluidics, the data were separated according to the lipid and solvent used. When ethanol was used, there was no obvious effect on either the particle size or PDI of liposomes composed of DSPC. However, at low concentrations, DOPC liposomes, while having a similar size, showed a relatively higher PDI. When IPA was used, there was no impact on particle size for DSPC or DOPC; however, again, DOPC liposomes showed a higher PDI at lower lipid concentrations. In the case of methanol, different concentrations of lipid were used depending on the production temperature. When comparing the concentrations at 20 °C, a low lipid concentration resulted in a smaller PDI and particle size for DSPC liposomes, whereas DOPC showed a slightly higher PDI and particle size at low concentrations. At 60 °C, the lipid concentration showed minimal effect on the particle size and PDI of DSPC liposomes, whereas DOPC liposomes showed a slightly higher particle size and PDI at low concentrations. Similar to using methanol in the production process, the initial concentration of the DSPC liposomes was determined by the production temperature. At 20 °C, the lipid concentration did not show any impact on the particle size, but the PDI was higher at the high concentration, whereas at 60 °C, the lipid concentration had no impact on the PDI or particle size. When DOPC was dissolved in Transcutol, the lipid concentration had minimal impact on the particle size and no effect on the PDI.

### 3.2. Exploring Interactions by Regression Trees

**Figure 3:**
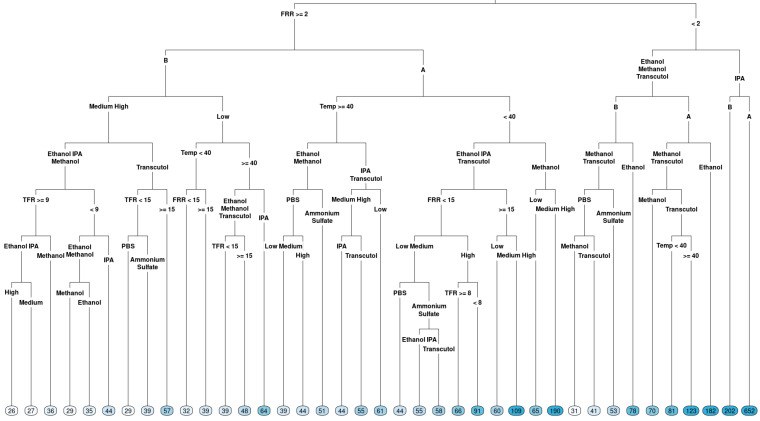
Regression tree for the particle size of liposomes produced via microfluidics following a DoE. Factors tested are shown in Table 1. Circles at the bottom represent predicted particle size, with darker colours indicating larger particle size.

**Figure 4:**
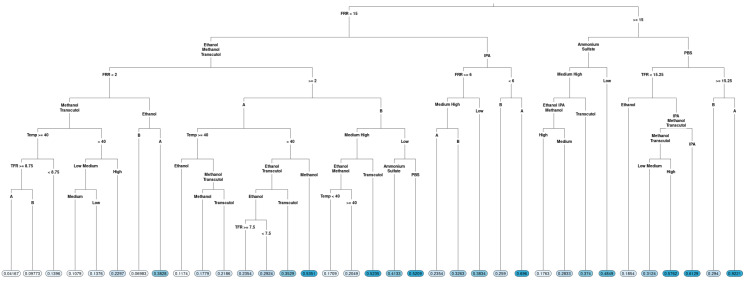
Regression tree of the polydispersity index (PDI) of liposomes produced via microfluidics following a DoE. Factors tested are shown in Table 1. Circles at the bottom represent predicted PDI, with darker colours representing larger PDI.

On top of boxplots, which focus on the overall effects, the regression trees were constructed for both measured outcomes (particle size and PDI) using the data collected to explore the interactions between factors. The regression tree can then be used to visualize how factors influenced either particle size (Figure 3) or PDI (Figure 4), where the first or ‘root’ node variable is the one that may have more of an impact on the response compared with the other factors tested. The splits indicate which variables would lead to important interaction effects. If a variable is not present in the tree, it means that its explanatory ability was limited in the given dataset for the response of interest. The terminal node on a graph shows the average response in the branch of the data following the split of a regression tree.

In terms of particle size (Figure 3), FRR was determined to be a root variable, which follows the observation in the boxplot: the right branch (FRR less than 2) had terminal nodes with a larger particle size, with the largest average particle size when the solvent IPA was used. A smaller particle size was observed for FRR less than 2 when methanol and Transcutol were used with lipid B (DOPC). Looking at the left-hand side of the tree, when FRR was greater than 2, a lower particle size was observed for lipid B compared to lipid A (DSPC). However, a lower particle size (averages between 39 and 61) was observed for temperatures higher than or equal to 40 °C. Temperature had a different effect direction for lipid B (DOPC) in low concentrations. Therefore, we cannot exclude the effect of temperature on the particle size fully, despite that we did not observe an overall effect. The complexity of the tree emphasizes that different factor combinations may potentially lead to the formation of liposomes with a lower particle size and some combinations lead to liposomes with large particle sizes.

In terms of PDI (Figure 4), similar to the particle size, the FRR was determined to be a root variable, with the threshold being a FRR 15:1, confirming boxplot visualization for the overall effect of FRR. In the next level, when the FRR was less than 15, the PDI was split according to the solvent used, and when the FRR was greater than or equal to 15, the PDI was split according to the buffer used, pointing out that interactions of FRR with the buffer and solvent were important for explaining PDI. Furthermore, liposomes with the lowest PDI were produced at FRR less than 2 from either methanol of Transcutol at temperatures below 40 °C when PBS was used as the buffer, and the TFR was greater than or equal to 13.25 mL/min (Figure 4, following the furthest left branches), indicating that higher-order interactions were present in the case of PDI.

Both regression trees point out that a statistical model fitting the particle size and PDI results would be complex and contain higher-order interactions and nonlinear (quadratic and even cubic) effects.

### 3.3. Modeling Results: Selected Factor Interactions

The model used to design the experiments was fitted using the collected data and had a good prediction performance. However, it was found to be overfitting the collected data, which was not generally observed in the fitted machine learning models (Supplementary Tables S2 and S3). Thus, Lasso regression (which was the selected final model) was then used to simulate the particle size and PDI across the experimental ranges defined in Table 1 to determine the combination of factors required for optimal liposome production.

In terms of particle size, Lasso regression determined that the mixing ratio (FRR), lipid concentration, and production temperature used were important factors in controlling the particle size, whereas the production speed, lipid, solvent, and the buffer used had no notable effect individually (Table 2), which confirmed the exploratory analysis provided in Section 3.1.

The mixing ratio (FRR) has been shown to be one of the key determinants of particle size [10,20,21], and these data not only demonstrated the factor’s importance individually but also showed how it could interact with other factors.

The particle size range could be fairly predicted when the interactions between FRR and either production speed (TFR), solvent, or lipid concentration were considered. Solvent has previously been shown to influence the particle size, with higher-polarity solvents resulting in smaller particles formed [15]. Webb et al. [15] reported that liposomes produced from methanol were significantly smaller than those produced from IPA. The work by Webb and co-workers suggested that this difference in particle size was driven by the rate of change in polarity of the solvents upon mixing with the aqueous buffer [15]. Higher-polarity solvents have a higher rate of change, which resulted in smaller disc formation and, therefore, smaller liposomes [15]. In a DoE study carried out by Lopez et al. [19], where the influence of organic solvents was assessed, specifically, methanol, ethanol, IPA, and Transcutol, on liposomes produced using a periodic disturbance mixer, Lopez and co-workers reported that Transcutol, the lowest-polarity solvent, produced the smallest liposomes when compared with ethanol, in contrast to the present study and the data reported by Webb and co-workers [15]. These contradicting results may demonstrate that the type of micromixer may affect the liposome size, as it would subsequently affect the rate of change of the polarity [19].

Lipid concentration was shown to have higher-order interactions with a number of factors, including the production speed, mixing ratio, lipid, buffer, and temperature. These interactions may be due to the variation in concentrations that were dependent on the lipid, solvent, and production temperature. The lipid concentration was shown to have an impact on the resultant liposome particle size; however, the type of lipid used was not selected as a model term. This suggested that the lipid concentration had a significant effect on the particle size, independent of the lipid used, and that the effect of the lipid concentration was similar for both lipids used.

Similarly, the lipid concentration was shown to effect PDI; however, the mixing speed and lipid were also found to impact the PDI of resultant liposomes (Table 3). When identifying two-way interactions, there were a couple of factors that together impacted polydispersity, whereas they had no effect individually. These interactions included the interaction between the mixing ratio and solvent (FRR:Solvent) and between temperature and solvent (Temp:Solvent).

Overall, when looking at all the selected model interactions (individual, two-way, three-way, and four-way), the factor that occurred the most for both particle size and PDI was the lipid concentration, closely followed by the TFR. This may be due to the varying solubility of the lipids in each solvent and, as previously mentioned, the self-assembly of lipids into liposomes is reliant on the diffusion rate of the solvent in the aqueous buffer, which is determined by the mixing speed. In the following section, we show the contour plots constructed based on the model simulations for further discussion on factor effects.

### 3.4. Contour Plots

To be delivered to their target site, nanocarriers must be of a small enough size to enter the target site and evade detection by the mononuclear phagocyte system (MPS). Therefore, nanocarriers are generally designed in the size range of less than 100 nm and low PDI, with smaller liposomes having longer circulation times (e.g., [22,23]) or distributing better into the relevant tissue [11]. Contour plots allow to check the regions where the final product would be in the desired range in terms of PDI and particle size.

**Figure 5:**
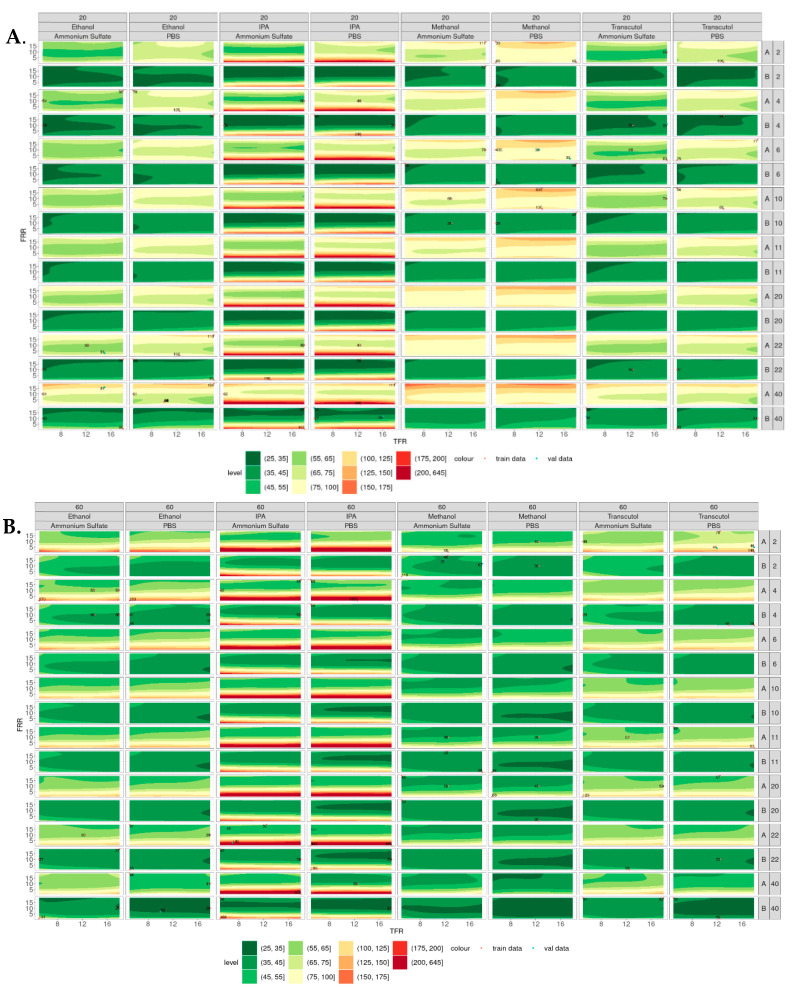
Contour plots of particle sizes produced across all the variables tested. (**A**) Liposomes produced at 20 °C from lipid A (DSPC) and lipid B (DOPC) at concentrations of 2–40 mg/mL. (**B**) Liposomes produced at 60 °C from lipid A (DSPC) and lipid B (DOPC) at concentrations of 2–40 mg/mL, where smaller liposomes are shown in green and larger liposomes are shown in red.

Figure 5 shows the contour plots of the simulated particle size of liposomes produced at 20 °C (Figure 5A) and 60 °C (Figure 5B), where the smaller particles appear green, and the larger particles appear red. In general, smaller liposomes were produced at 60 °C compared with 20 °C, with IPA showing the largest variability in size across all temperatures, lipid and buffer concentrations. Liposomes produced from IPA at low FRR (e.g., 1:1) at all TFR tested showed relatively larger particle sizes compared with the other solvents tested. This may be due to the polarity of this solvent, as solvents with lower polarity (e.g., IPA) result in larger liposomes than high-polarity solvents (e.g., methanol). This is due to the low-polarity solvents having a lower rate of change in polarity when mixed with the aqueous phase, leading to larger lipid disc formation and, therefore, larger liposome formation [15]. As the concentration of the lipid increased, the size of the liposomes increased (at 20 °C).

**Figure 6:**
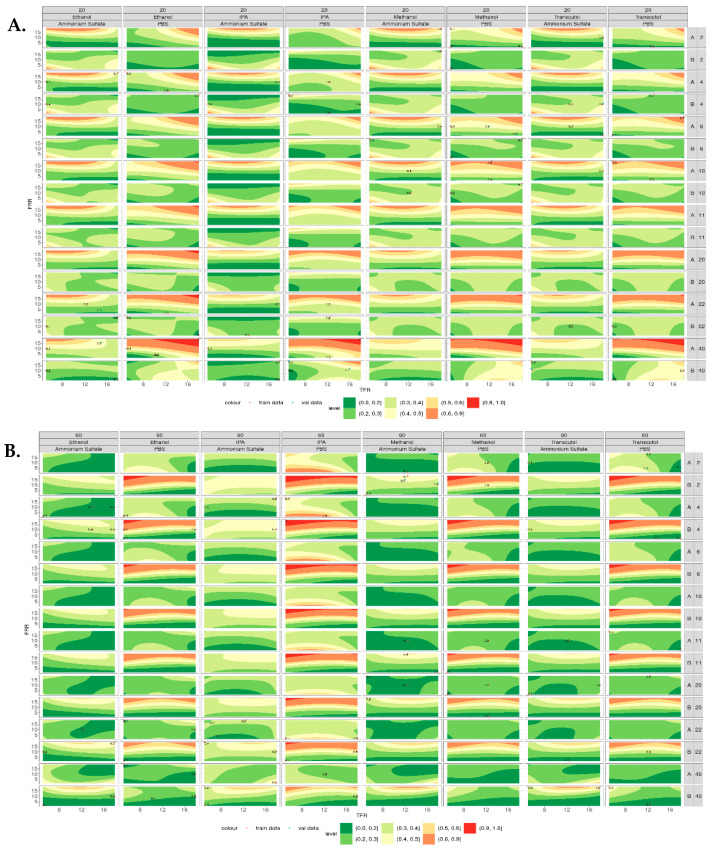
Contour plots of the polydispersity index of liposomes produced across all the variables tested. (**A**) PDI of liposomes produced at 20 °C from lipid A (DSPC) and lipid B (DOPC) at concentrations of 2–40 mg/mL. (**B**) PDI of liposomes produced at 60 °C from lipid A (DSPC) and lipid B (DOPC) at concentrations of 2–40 mg/mL, where lower PDI appears green and higher PDI appears red.

Figure 6 shows the contour plots for the polydispersity index (PDI) of the liposomes produced at both 20 °C (Figure 6A) and 60 °C (Figure 6B). When produced at 20 °C, liposomes composed of DSPC showed an increase in PDI with the increasing lipid concentration when PBS was used as the aqueous buffer; however, this was not observed when ammonium sulfate was used. When ammonium sulfate was used as the aqueous buffer, the PDI of DSPC liposomes decreased with the increasing lipid concentration. This trend can be observed across all the solvents tested. Liposomes composed of DOPC showed a similar trend, with liposomes produced using PBS having increased PDI with an increased lipid concentration, whereas those produced using ammonium sulfate showed decreased PDI with the increasing lipid concentration. Again, this was observed across all the solvents tested. However, when DSPC and DOPC liposomes were produced at 60 °C (Figure 6B), the opposite trend was observed. When DOPC was produced in PBS, the PDI decreased as the lipid concentration increased, whereas DSPC did not appear to change as the concentration increased.

### 3.5. Confirmatory Experiments

**Figure 7:**
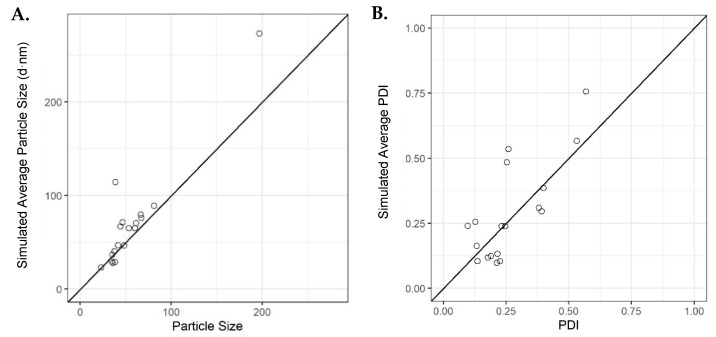
Average simulations for the validation runs using the initial model for (**A**) particle size versus observed and (**B**) PDI versus observed. Solid dashed line is an identity line.

Verification runs were then carried out to confirm the average simulated outcomes of the initial model using the original dataset. Eighteen runs were selected, where the average simulated values were ranging from low to high, specifically (23, 273) and (0.097, 0.755) for PSD and PDI, respectively. The results from these experiments showed that the initial model accurately simulated the majority of the particle sizes and few of the PDIs for some of the runs carried out; however, there were results that deviated from the simulated outcomes based upon the model obtained through machine learning (Figure 7). These deviations may be due to the low final lipid concentration used when measuring the liposomes and the high complexity of the model. This is supported by some initial investigations performed (see the Supplementary Materials), where we showed that at lipid concentrations below 1 mg/mL, there can be significant deviations in both size and PDI compared with samples of higher concentrations (Supplementary Figure S5).

## 4. Discussion

Microfluidic production of liposomes may provide a cost-effective and scalable approach to the manufacturing of liposomes. Since the first documented application of microfluidics for the production of liposomes by Jahn et al. [8], research has focused on how this technology can be developed and fine-tuned to produce a variety of liposomes for drug delivery.

Extensive research has demonstrated the impact of microfluidic parameters, including the mixing ratio, or flow rate ratio (FRR), and the production speed, or total flow rate (TFR), on the critical quality attributes of the liposomes, such as particle size and particle size distribution. Of these, the most notable parameter affecting liposome size was the mixing ratio (FRR), which has been shown to have a significant impact on the liposome size, independent of the production speed and formulation tested in the present work. FRR affects the rate at which the organic phase disperses within the aqueous phase; therefore, at a higher FRR, there is a larger proportion of aqueous buffer, allowing for faster dispersion of the organic phase, resulting in a shorter period for lipid disc formation and subsequently resulting in smaller particle formation [15]. Increasing the FRR resulted in larger particle formation, as shown in Figure 1A,B, whereas a lower FRR (1:1) resulted in significantly larger particle sizes compared with FRR > 10:1. This difference in size may be due to the dilution rate of the solvent in the aqueous buffer, as also previously suggested by Lopez and coworkers [19]. As the aqueous phase and the organic phase mixed together, the alcohol concentration was diluted into the aqueous buffer. Therefore, increasing the FRR resulted in an increased aqueous volume and increased solvent dilution, thereby promoting faster mixing, resulting in the production of smaller liposomes [24].

The formation of liposomes was, therefore, also dependent on the change in polarity of the solvents [15]. During microfluidic production, the lipids were initially dissolved in an organic solvent, which was then mixed with the aqueous buffer to promote self-assembly of the liposomes. As previously mentioned, it was the reduction in polarity and diffusion of the solvent into the aqueous buffer that drove liposome formation. Therefore, these data suggested that reducing the polarity of the solvent will result in an increased liposome size. This was demonstrated when comparing liposomes produced from IPA and those produced from Transcutol. The water miscibility of a solvent is correlated with the carbon chain length and the surface tension. As the carbon chain increases, the polar group becomes a smaller fraction of the overall chemical composition and, therefore, results in decreased solubility and polarity [15]. Transcutol had the longest carbon chain length (14 carbons) of the four solvents selected (Supplementary Table S4) and, therefore, will have the lowest polarity, resulting in the largest particle size. As mentioned previously, research carried out by Webb et al. [15] showed that liposomes produced from either methanol or ethanol were of similar size; however, there was a noticeable increase in size when IPA was used.

Before selecting the solvent, there are several other factors that must be considered, such as the solubility of the lipid in the selected solvent and the aqueous phase. As mentioned above, increasing the solvent polarity resulted in smaller liposome formation; however, Joshi et al. also suggested that the sensitivity to this polarity change may be lipid-dependent [24]. When changing from methanol to ethanol, there was no notable difference in the size of liposomes composed of PC, DMPC, or DPPC; however, those composed of DSPC:cholesterol showed a significant increase in particle size. Work carried out by Zook et al. also demonstrated how the length of the alkyl chain and transition temperature of the lipid affect particle formation. These studies showed that longer alkyl chain length/high transition temperature lipids had a higher elasticity modulus and, therefore, a more rigid membrane when formed at or below the transition temperature, allowing for larger particle formation [25]. The lipids selected here both had acyl chain lengths of 18 carbons; however, DSPC is saturated whereas DOPC is unsaturated. The same sensitivity was also shown when switching the buffer from PBS to Tris buffer [24]. The initial lipid concentration is another important consideration when preparing liposomes, as it has been shown that increasing the initial lipid concentration results in smaller vesicle formation, irrespective of the alkyl chain length [18,24]. In order to determine whether this difference in particle size was due to the lipid alkyl chain length or the lipid transition temperature, Forbes et al. [18] compared the particle size of DSPC and DOPC:cholesterol at a constant TFR and FRR and at the same initial lipid concentration. It was shown that DOPC liposomes were significantly larger than DSPC liposomes, which is in disagreement with the results presented here; however, it is important to consider that the liposomes produced in this study had the addition of DSPE-PEG2000 in the bilayer. The addition of PEG polymers to the liposome surface reduces opsonization and clearance by the immune system, therefore, extending their circulation in the blood stream [26]. Increasing the concentration of PEG has been shown to result in a decrease in liposome size [27], therefore, accounting for the difference in size.

When produced by conventional manufacturing methods, liposomes have to be manufactured at temperatures above the transition temperature of the main lipid. The lipids would initially be dissolved in a solvent and undergo evaporation, before then being rehydrated in the desired aqueous buffer. During the rehydration stage, the lipids are heated to change the structure of the lipid from a crystalline structure to a more fluid gel phase, allowing for the lipid disc to bend and curve to form vesicles. However, microfluidics can produce small, monodisperse liposomes at temperatures below the transition temperature. There was no notable difference in particle size of liposomes produced at 20 °C and 60 °C; however, statistical analysis showed that temperature was a significant factor when considered in combination with other production parameters. This may be attributed to the bottom-up manufacturing approach, where the liposomes were produced from lipid monomers, which self-assemble into vesicles in the presence of an aqueous buffer. Whereas in conventional top-down approaches, the liposomes are produced and then undergo subsequent size reduction steps, in which the liposomes are broken down and reformed [18]. To form curved vesicles, these lipids need to be heated to make them more flexible and fluid.

Further analysis of the data presented in the present study provided insight into how microfluidic and formulation parameters can interact to determine the resultant liposome size and polydispersity. Similar DoE studies have been carried out to demonstrate the interaction between various liposomes production parameters, including TFR, FRR, and solvent; however, none have previously investigated as many factors or as wide a range as the present DoE. Kastner et al. [13] carried out a microfluidic-based study to assess the impact of FRR and TFR using multivariate analysis. In the study by Kastner and co-workers, cationic liposomes were manufactured at FRR 1:1–5:1 and at TFR 0.5–2 mL/min, and it was found that increasing the FRR resulted in a decreased particle size and TFR had no significant effect. These results confirmed previous data [10,20] as well as the data presented in this study. It was also shown that increasing the FRR had a significant impact on PDI. This aligns with the results in this study, as increasing the FRR from 1:1 to 19:1 resulted in a steady increase in PDI (Figure 5).

Transcutol, more commonly known as diethylene glycol monoethyl ether (DEGEE), was originally used as an industrial solvent under the trade name Carbitol and, up until the 1990s, was not suitable for pharmaceutical application due to the high level of impurities, such as ethylene glycol [28]. Transcutol is an ethylene oxide derivative with high purity that is used commercially in over-the-counter topical products due to its ability to enhance skin penetration [28,29]. Recent research carried out by Lopez et al. investigated the use of Transcutol for liposome production [19]. In these experiments, DMPC:cholesterol:DHP liposomes were produced using a periodic disturbance mixer using four different solvents: methanol, ethanol, IPA, and Transcutol. These liposomes were initially produced at a constant TFR, FRR, and initial lipid concentration, and it was shown that liposomes produced from IPA had a larger PDI, and Transcutol produced the smallest liposomes. These results agree with the results presented here; however, Lopez et al. found that Transcutol and IPA produced smaller liposomes than ethanol and methanol. These contradicting results may be due to the different types of micromixer. The design of the micromixer influences the mixing and subsequent dilution of the organic solvent in the aqueous buffer and, therefore, different designs may result in different mixing efficiencies [19]. It is important to consider that the model produced here used only one type of mixer and a small range of lipid formulations, as the key aim of this study was to model the manufacturing of liposomes on this specific system (NanoAssemblr Benchtop). The microfluidic cartridge employed here was a staggered herringbone micromixer that relied on laminar flow and passive mixing. Therefore, the results here would predominantly apply to this type of microfluidic mixing, and another model would need to be developed using active mixing and/or turbulent flow mixers in order to fully demonstrate how microfluidic parameters and manufacturing of different mixers can affect the resultant production of liposomes. Further experiments or validation runs could be carried out on another type of laminar, passive microfluidic mixer to identify whether the results here can be applied to other related mixers.

It was also shown that the production temperature and resultant size of the liposomes may be dependent on the solvent used. Transcutol and ethanol were compared at four different temperatures, and it was shown that with the increasing temperature, the size of the liposomes produced from ethanol decreased, whereas the liposomes produced from Transcutol showed no effect. In the present study, it was shown that temperature alone had no overall effect on the resultant liposome physicochemical characteristics; however, further analysis showed that temperature can interact with the lipid concentration and lipid and solvent used to determine both the size and PDI.

With the number of factors being investigated in this study, it was important to find a method that could screen all the variable without having to change one factor at a time and try every combination. High-throughput screening methods allow for the rapid analysis of large quantities of data while minimizing costs. There are a number of different ways to rapidly screen a large variety of compounds or factors, and some of these include fluorescent labels, which can be used to tag different cells and proteins. These signals can then be detected via microscopy or flow cytometry, and the different fluorescence corresponds to different proteins [30].

In order to provide a full detailed analysis of the factors important for microfluidic production of nanoparticles, a design of experiments (DoE) approach was selected. DoE is a systematic, statistical approach with the aim of exploring and modeling multiple responses across multiple factors of a given design space [31]. Confirmatory experiments were carried out to help validate the model developed in this DoE, and the results from this demonstrated that the model was able to accurately determine the liposome characteristics of five out of the eight runs carried out (approximately 63% accuracy). The error in the simulated output may come from higher-order interactions that we were not able to determine with the current model due to the number and wide range of variables tested in this study.

In line with previous research and design of experiment studies investigating microfluidic production, our study provided further in-depth statistical analysis of not only process but also formulation parameters, and how these influence the liposome physicochemical characteristics. Compared with previous research, the present study investigated a larger number of manufacturing factors and a wider range of manufacturing parameters. Through this, we have tested the limits of the NanoAssemblr benchtop system and demonstrated the ability of microfluidics to produce a wide range of liposomes under various production conditions.

## 5. Conclusions

Our studies have demonstrated the versatility and ability of the microfluidic platform to produce liposomes of various compositions and particle sizes. Design of experiments analysis confirmed the statistical significance of both microfluidic process parameters and formulation parameters on the resultant liposome particle size and polydispersity. The complexity of the data generated by the DoE required machine learning to effectively model the results. The model suggested that the resultant particle size and PDI can be adjusted through the tuning of the formulation, such as the lipid and solvent used, as well as through changing the mixing ratio (FRR) and production speed (TFR); however, it was only possible to simulate directions of particle size and PDI with the generated models, not to accurately simulate the liposome size and PDI. This demonstrates the complexity of liposome formulation and manufacturing and the large connectivity between the factors.

## Figures and Tables

**Table 1:** Factors investigated and range tested in the DoE study.

Factor	Range
Total flow rate (TFR)	5, 10, 12.5, 18 mL/min
Flow rate ratio (FRR)	1:1, 3:1, 10:1, 19:1
Lipid concentration	2–40 mg/mL
Lipid	DSPC, DOPC
Solvent	Ethanol, Methanol, IPA, Transcutol
Buffer	Phosphate-buffered saline (PBS), ammonium sulfate
Temperature	20 °C, 60 °C

**Table 2:** Selected model terms for the particle size of liposomes prepared using microfluidics using Lasso regression.

Model Term (PSD)
FRR
LipidConc
Temp
TFR2
FRR:LipidConc
FRR:Solvent
FRR:TFR2
LipidConc:Lipid
LipidConc:LipicConc2
Temp:Lipid
Temp:Solvent
Temp:LipidConc2
Lipid:TFR2
Buffer:TFR2
Solvent:TFR2
TFR2:LipidConc2
TFR:Temp:TFR2
TFR:Lipid:TFR2
TFR:Buffer:TFR2
FRR:LipidConc:LipidConc2
FRR:Buffer:Solvent
FRR:Solvent:TFR2
LipidConc:Temp:FRR2
LipidConc:Temp:LipidConc2
Temp:Lipid:Buffer
Temp:Lipid:Sovent
Buffer:TRF2:FRR2
TFR:FRR:LipidConc:LipidConc2
TFR:FRR:Temp:LipidConc2
TFR:LipidConc:Temp:Lipid
TFR:LipidConc:Temp:TFR2
TFR:LipidConc:FRR2:LipidConc2
TFR:Temp:Lipid:Solvent
FRR:LipidConc:Temp:LipidConc2
FRR:LipidConc:Lipid:Buffer
FRR:LipidConc:Lipid:Solvent
FRR:LipidConc:FRR2:LipidConc2
FRR:Temp:Lipid:Solvent
FRR:TFR2:FRR2:LipidConc2
LipidConc:Temp:Lipid:TFR2
LipidConc:Temp:Buffer:TFR2
LipidConc:Temp:TFR2:FRR2
LipidConc:Lipid:TFR2:FRR2
Temp:Lipid:Buffer:Solvent
Temp:Lipid:Buffer:FRR2
Temp:Solvent:FRR2:LipidConc2
Lipid:Buffer:Solvent:LipidConc2
Buffer:Solvent:FRR2:LipidConc2

**Table 3:** Selected model terms for PDI using Lasso regression.

Model Term
TFR
LipidConc
Lipid
TFR:FRR
FRR:Solvent
LipidConc:LipidConc2
Temp:Solvent
Temp:LipidConc2
Solvent:LipidConc2
Solvent:TFR2
TFR:FRR:Solvent
TFR:LipidConc:Lipid
TFR:LipidConc:TFR2
TFR:Temp:Lipid
TFR:Temp:FRR2
TFR:Lipid:TFR2
TFR:FRR2:LipidConc2
FRR:LipidConc:Temp
FRR:LipidConc:TFR2
FRR:LipidConc:LipidcConc2
FRR:Buffer:Solvent
FRR:Solvent:LipidConc2
LipidConc:Temp:FRR2
LipidConc:Lipid:Solvent
LipidConc:Lipid:TFR2
LipidConc:Lipid:FRR2
Temp:Lipid:Solvent
Lipid:Solvent:TFR2
Buffer:Solvent:TFR2
TFR:FRR:LipidConc:Temp
TFR:FRR:LipidConc:Lipid
TFR:FRR:Temp:LipidConc2
TFR:FRR:Lipid:LipidConc2
TFR:FRR:Solvent:LipidConc2
TFR:Temp:Lipid:Buffer
TFR:Temp:Buffer:TFR2
TFR:Temp:Buffer:FRR2
TFR:Temp:FRR2:LipidConc2
TFR:Lipid:Buffer:TFR2
TFR:Buffer:Solvent:TFR2
FRR:LipidConc:Temp:Lipid
FRR:LipidConc:Temp:Solvent
FRR:LipidConc:Temp:LipidConc2
FRR:LipidConc:FRR2:LipidConc2
FRR:Lipid:Buffer:Solvent
LipidConc:Temp:Buffer:TFR2
LipidConc:Temp:Buffer:FRR2
LipidConc:TFR2:FRR2:LipidConc2
Temp:Lipid:Buffer:Solvent

## Data Availability

The data underpinning this publication are openly available from the University of Strathclyde KnowledgeBase at: https://doi.org/10.15129/f1aff828-eed2-4c52-93a9-b71df5fbee22.

## Supplementary Materials

The following supporting information can be downloaded at: https://www.mdpi.com/article/10.3390/pharmaceutics16091159/s1, Figure S1: Results of cross validation for shrinkage and mixing parameters selection for log(PSD); Figure S2: Simulations of the final model for PSD d50 (on log scale) versus observed: green solid dots are results for validation set, black circles are results for training set; Figure S3: Results of cross validation for shrinkage and mixing parameters selection for logit(PDI); Figure S4: Simulations of the LASSO model for PDI (on logit scale) versus observed: green solid dots are results for validation set, black circles are results for training set; Table S1: Comparative study results for different predictive models—PSD; Table S2: Comparative study results for different predictive models—PDI; Table S3: Details of the different total lipid concentrations used for each of the selected phospholipids in the selected solvents at either 20°C or 60°C. The final lipid composition consisted of DSPC/DOPC:Cholesterol:DSPE-PEG2000 (*w*/*w* 3:1:1); Table S4: Chemical properties of the selected solvents; Figure S5: Sensitivity of Malvern Zetasizer. DSPC:Chol:DSPE-PEG2000 liposomes were measured at varying concentrations for size, PDI, Derived count rate and attenuation. (A) Shows one batch of liposomes measure at concentrations from 0.05–10 mg/mL, (B) shows a second batch of liposomes measured at concentrations from 0.05–10 mg/mL. Demonstrates that the zetasizer is sensitive and accurate at concentrations > 0.2 mg/mL. Each figure represents n = 1.
