# Supplementary material for: Can We Simplify Liposome Manufacturing Using a Complex DoE Approach?
Source: Pharmaceutics. 2024 Sep 1;16(9):1159. doi: 10.3390/pharmaceutics16091159 (PMC11435235; doi:10.3390/pharmaceutics16091159)
Supplement: Supplementary file 1 [file pharmaceutics-16-01159-s001.zip › pharmaceutics-3158262-supplementary.pdf]

Statistical methodology for data analysis

1.1. DoE

The study design involved setting up an I-optimal DoE (A) with 135 experimental runs including control runs to check the reproducibility of the process and measurements. The DoE has a possibility to estimate several third-order interaction terms and fourth order interaction term.
Additionally, 18 validation runs were created to check how well the model simulates the PSD and PDI on new combinations of factor settings which were not used in the first DoE.

1.2. Statistical modeling

As the current dataset was obtained via I-Optimal DoE, the multiple linear regression models as well as regularized linear regression models (such as Elastic Net and Lasso) were considered. The primary linear regression model had very good performance on the training set and high error on the validation set of experiments, the phenomenon known in statistical data analysis as overfitting. To overcome the overfitting, regularized linear models were employed. Application of regularized models allows to avoid overfitting and perform model selection at the same time, as the least relevant model terms would be set to zero. The resulting simulation error of regularized regression using Elastic Net was higher compared to the original multiple linear regression model, however, the simulation error was comparable for training and validation set, which justifies its use from the simulation perspective. Additional modeling efforts have included machine learning methods, such as Random Forests and XGBoost but their performance in terms of simulation error was not better compared to the Elastic Net models.

Full technical details on the modeling are provided in Supplementary Information.

1.3. Visualization of modeling results

Due to complexity of DoE and the final selected models involving higher order interactions which were relevant for simulation, contour plots were constructed for ease of interpretation. A typical contour plot would display the average response surface in terms of continuous factors TFR and FRR for different combinations of categorical factors solvent, buffer, lipid and discretized lipid concentrations and temperatures. Contour plots allow to visualize and put emphasis on the complex interactions, however the model uncertainty (i.e. average simulation error) is not taken into account. In addition, the measured data points from training set and validation set were added to the contour plots to demonstrate how the final selected model fits measurements from both sets of results.

2. Supplementary information: Statistical methodology

2.1. Data preparation

The main dataset (referred to as training dataset) consists of 135 DOE runs. Additionally, 18 validation runs were generated to check how well the model simulates the PSD and PDI on new observations which were not used in the model.

As the main dataset was created according to the DoE, the construction of simulator matrix X had to include not only main effects of the seven considered factors (TFR, FRR, Temperature, Lipid Concentration, Lipid, Solvent, Buffer), but also their interactions. The original design had a possibility to estimate several third-order interaction terms and fourth-order interaction term, but after collecting the data it became clear that more complex model may be needed with additional higher order interaction terms. Since these additional higher order interaction terms are highly correlated with the model terms in original DoE, model selection was necessary. The classical approaches for multiple linear regression such as stepwise procedure was not able to suggest a good predictive model which would perform well both on training set and on the validation data.

The continuous DoE factors TFR, FRR, Temperature and Lipid Concentration were normalized to the range [-1; 1] by equation $X_{norm} = 2 * (X - (X_{min} + X_{max})/2)/(X_{max} - X_{min})$. The Lipids were encoded as 1 in case of Lipid A and -1 in case of Lipid B, the Buffers were encoded as 1 for PBS and -1 for Ammonium Sulfate. For solvents, the set of three "dummy" 0-1 variables were constructed: Solvent EtOH = 1 if EtOH was used, and 0 otherwise, Solvent IPA=1 if IPA was used and 0 otherwise, Solvent MeOH = 1 if MeOH was used and 0 otherwise.
The factors in the validation data were also normalized using the same ranges as the training set to avoid potential bias due to normalization. This normalization to [-1;1] scale allows for less correlation between main effects and quadratic terms and at the same time give equal weight to all model terms. Subsequently, no additional normalization is required during modeling.

Final set of normalized predictors (matrix X) contained main effects of all experimental factors, quadratic effects of TFR, FRR and actual Lipid concentration, the interactions included up to the 4th order of these factors. In this way terms like quadratic TFR effect, quadratic FRR effect with the combination of any other two factors would be considered and even result in higher order effects. For instance, $TFR^2 * TFR$ would be third order term for TFR included in the model.

The responses, PSD and PDI were also transformed prior to analysis due to the fact that PSD can only be positive and PDI can be only between (0 and 1): log(PSD) was used for the former and logit(PDI)=log(PDI/(1-PDI)) for the latter response.

The construction of model matrix is also a part of R script provided for reproducibility purpose

2.2. Modeling by regularized linear models

2.2.1. Method description

The family of regularized linear models consists of Ridge regression, LASSO (least absolute shrinkage and selection operator) and the generalization Elastic Net (B). These methods essentially are linear regression models, which on one hand can work with multiple model terms which are highly correlated (primary goal of Ridge regression) and on the other hand they can perform selection of model terms most relevant for simulation by shrinking the least important model terms to zero. Unlike multiple linear regression models, which are based on ordinary least squares estimates and do not have any specific parameters which should be selected, the regularized linear models have additional parameters, such as amount of shrinkage, λ in case of ridge regression and LASSO and extra parameter α for Elastic Net, which would allow to determine the balance between model selection (LASSO) and inclusion of correlated terms (Ridge regression).

As the dataset was generated using DOE, in order not to destroy the structure leave-one-out cross validation scheme was used.

The modeling was performed in R4.0.3 using the packages caret_6.0-86 and glmnet_4.0-2.

2.2.2. Cross validation results

2.2.2.1. PSD model

Figure S1 shows the results for selection of the regularization λ and mixing parameters α, for modelling of PSD, suggesting that Lasso models have the lowest RMSE (α=1), which allows to make a parsimonious model. The optimal regularization parameter, λ, was chosen to be 0.01568802.

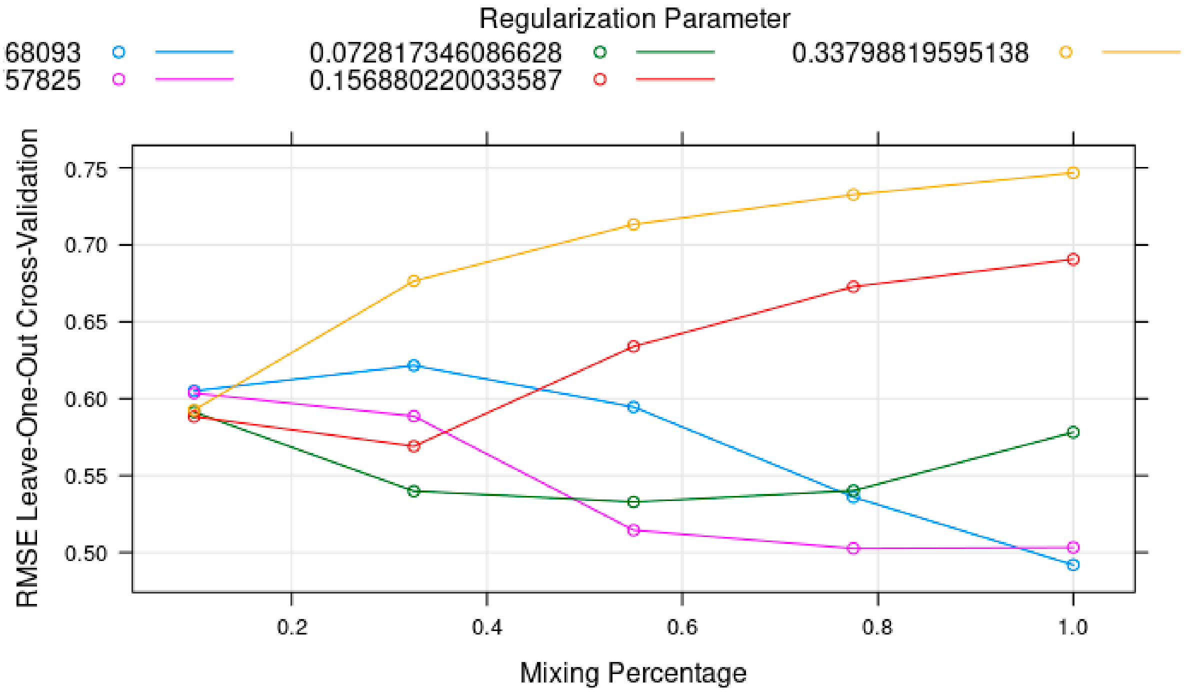

Figure S1: Results of cross validation for shrinkage and mixing parameters selection for log(PSD).

The final model for log(PSD) of 50 terms, which are shown in the supplementary HTML document.

Figure S2 shows the results of simulations against the observed PSD D50 for both training and validation datasets, demonstrating comparable simulation error.

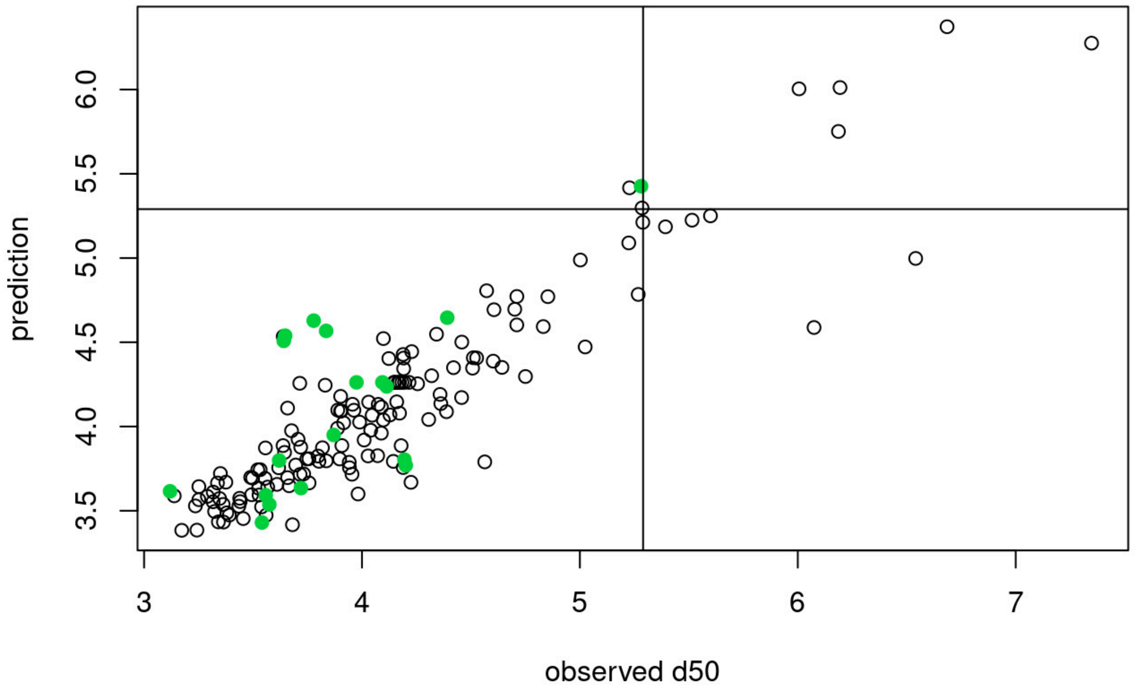

Figure S2 – Simulations of the final model for PSD d50 (on log scale) versus observed: green solid dots are results for validation set, black circles are results for training set.

2.2.2.2. PDI model

Figure S3 shows the results for selection of the regularization λ and mixing parameters α, for modelling of PDI, and unlike for PSD, here suggesting that models closer to Ridge Regression have the lowest RMSE (α=0.1). The model is also more complex compared to the model of PSD and consists of 273 terms. In the end, we have also used LASSO model, to sacrifice a part of simulation error towards the parsimony of the model. For LASSO the optimal shrinkage value λ was set to 0.05226027. The model selection by LASSO resulted in 51 model terms, out of which 14 terms were common with the selected model of PSD.

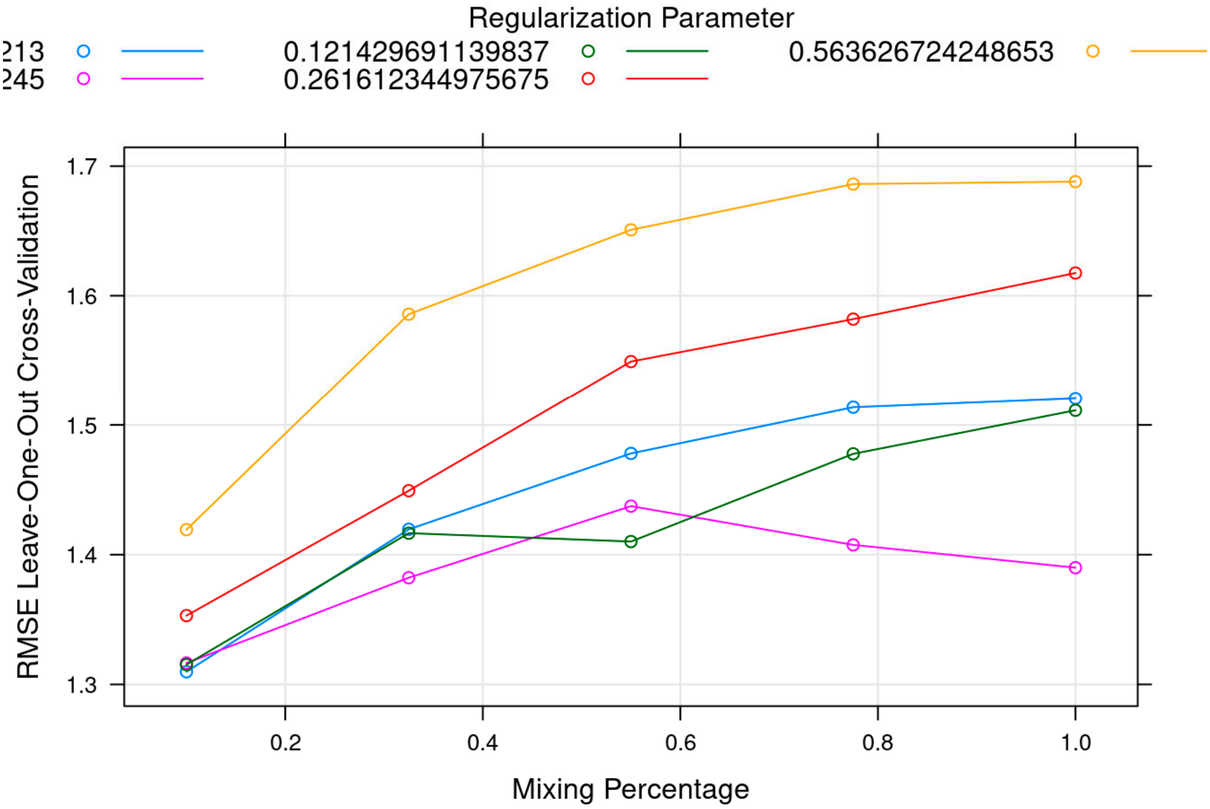

Figure S3 - Results of cross validation for shrinkage and mixing parameters selection for logit(PDI).

Figure S4 shows the results of simulations against the observed PDI for both training and validation datasets, demonstrating comparable simulation error.

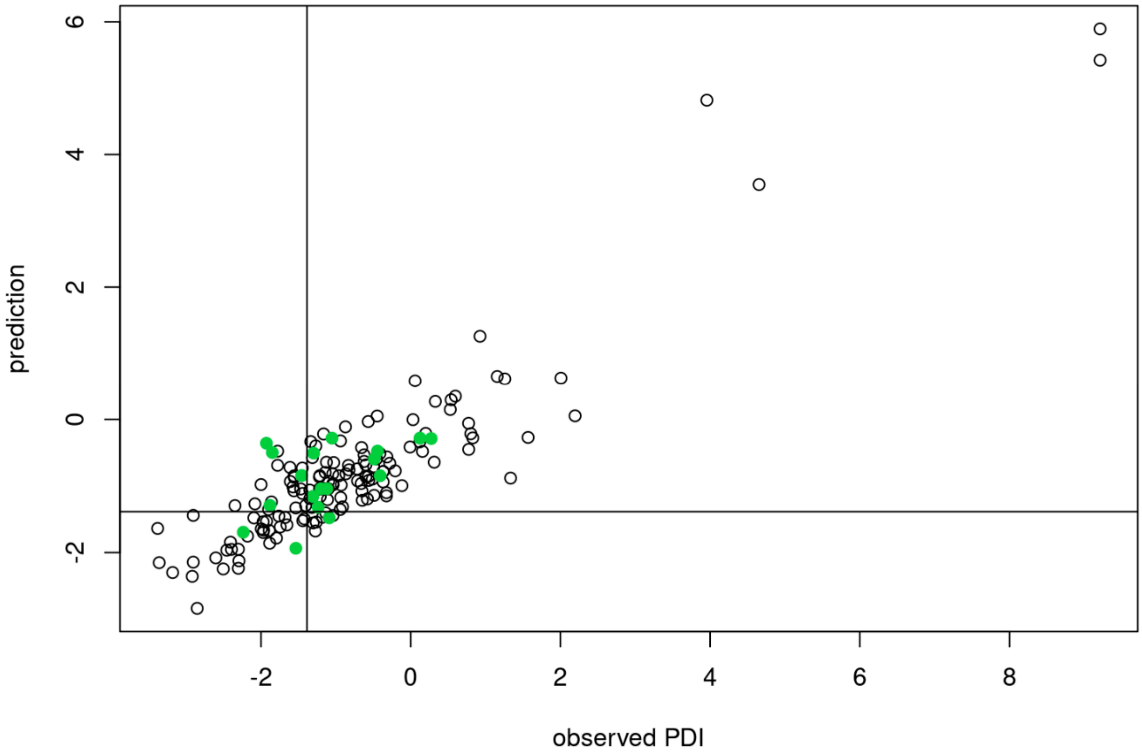

Figure S4 – Simulations of the LASSO model for PDI (on logit scale) versus observed: green solid dots are results for validation set, black circles are results for training set.

2.3. Comparison of regularized linear regression models to the machine learning methods

In order to attempt finding a better predictive model which would do better than regularized linear regression on both training and validation data, a comparative study has been conducted involving several machine learning methods, such as random forests, XGboost, MARS, PLS and QRF. Here we briefly summarize the methods.

Random forest (C) is a non-parametric predictive modeling approach where a set of regression tree is constructed based on bootstrapping the data. In general, it does not require specification of complex higher order interactions in predictor matrix X, like in linear models, as the regression trees handle complex interactions while modeling the data.

XGboost (D) is a scalable end-to-end tree boosting system which is used widely by data scientists to achieve state-of-the-art results on many machine learning challenges. It is an implementation of gradient boosted decision trees designed for speed and performance in form of software library that you can download and install on your machine, then access from a variety of interfaces.

Like neural networks and partial least squares, MARS (E) uses surrogate features instead of the original predictors. However, whereas PLS and neural networks are based on linear combinations of the predictors, MARS creates two contrasted versions of a predictor to enter the model. Also, the surrogate features in MARS are usually a function of only one or two predictors at a time. For MARS models that can include two or more terms at a time, we have observed occasional instabilities in the model simulations where a few sample simulations are wildly inaccurate (perhaps an order of magnitude off of the true value). This problem has not been observed with additive MARS models.

PLS is a commonly applied method in chemometrics which tries to find optimal predictive model by maximizing covariance between predictors and response. It requires setting of the number of components that should be used for prediction, which should be selected by cross validation. When the number of components gets to its maximum (here, to the number of experiments in the dataset), the solution corresponds to the ridge regression.

Quantile Random Forest (F) also called Quantile Regression Forests, give a non-parametric and accurate way of estimating conditional quantiles for high-dimensional predictor variables.

Table S12 shows the results of the comparative study for PSD. The LASSO models have very reasonable performance both on training and test set and involvement of more sophisticated machine learning methods did not improve the performance considerably. We can see that XGboost outperforms the rest on the training set, but its performance on the test set was comparable to the LASSO one. Therefore, to work with models which are easier to interpret, we concluded that LASSO would be the optimal choice.

Table S1 – Comparative study results for different predictive models - PSD.

ModelName	RMSE.train	RMSE.test	Rsq.train	Rsq.test
lassoTuned	0.363	0.309	0.799	0.693
elasticNetTuned	0.361	0.326	0.801	0.659
rfTuned	0.207	0.297	0.936	0.643
xgbTuned	0.007	0.281	1.000	0.628
gbmTuned	0.310	0.297	0.839	0.578
marsTuned	0.401	0.404	0.707	0.437
ridgeTuned	0.385	0.392	0.842	0.259
plsTuned	0.393	0.414	0.719	0.192
qrfTuned	0.701	0.448	0.221	0.044

Below Table S23 shows results of the comparative study for PDI.

Table S2 - Comparative study results for different predictive models - PDI

ModelName	RMSE.train	RMSE.test	Rsq.train	Rsq.test
lassoTuned_PDI	0.190	0.778	0.988	0.189
elasticNetTuned_PDI	0.377	0.654	0.960	0.171
plsTuned_PDI	0.442	0.705	0.931	0.165
ridgeTuned_PDI	0.667	0.664	0.899	0.112
xgbTuned_PDI	0.012	0.829	1.000	0.108
rfTuned_PDI	0.791	0.867	0.881	0.018
qrfTuned_PDI	1.649	0.679	0.119	0.010
marsTuned_PDI	1.531	1.188	0.166	0.006
gbmTuned_PDI	0.620	1.072	0.883	0.000

2.4. Selection of Lipid Concentration

Table S3: Details of the different total lipid concentrations used for each of the selected phospholipids in the selected solvents at either 20°C or 60°C. The final lipid composition consisted of DSPC/DOPC:Cholesterol:DSPE-PEG2000 (w/w 3:1:1).

Solvent	Lipid	20°			60°C		
		Low (mg/mL	Medium (mg/mL)	High (mg/mL)	Low (mg/mL	Medium (mg/mL)	High (mg/mL)
Methanol	DSPC	2	6	10	2	11	20
	DOPC	2	6	10	2	11	20
Ethanol	DSPC	4	22	40	4	22	40
	DOPC	4	22	40	4	22	40
IPA	DSPC	4	22	40	4	22	40
	DOPC	4	22	40	4	22	40
Transcutol	DSPC	2	6	10	2	11	20
	DOPC	4	22	40	4	22	40

2.5. Solvent Properties

Table S4: Chemical properties of the selected solvents.

	Methanol	Ethanol	IPA	Transcutol
MW (g/mol)	32.042	46.07	60.1	134.17
Chemical Formula	CH_3OH	C_2H_5OH	C_3H_8O	$C_6H_{14}O_3$
Melting Point (°C)	-97.6	-114.1	-89.5	-76
Boiling Point (°C)	64.7	78.2	82.3	196
Flash Point (°C)	9.7	14	12	96
LogP	-0.77	-0.31	0.05	-0.54
Viscosity (mPa.s)	0.544	1.074	2.038	3.85
pKa	15.3	15.9	17.1	14.37

2.6. Size and PDI SOP Optimisation

Before beginning the Design of Experiments project it was important to determine the sensitivity of the Malvern Zetasizer to different concentrations of liposomes. To do this, DSPC:Chol:PEG2000 liposomes were produced from a 40 mg/mL stock solution in ethanol at FRR 3:1 and TFR 10 mL/min with a final lipid concentration of 10 mg/mL. These liposomes then underwent TFF purification before being diluted in filtered PBS to the desired concentrations for sizing. The parameters for the sizing protocol are shown in section 2.3. The results show that at lower concentrations (<0.2 mg/mL) there is an increase in variation of both size and PDI which is corrected when the sample is left to stabilise and remeasured (Figure S5).

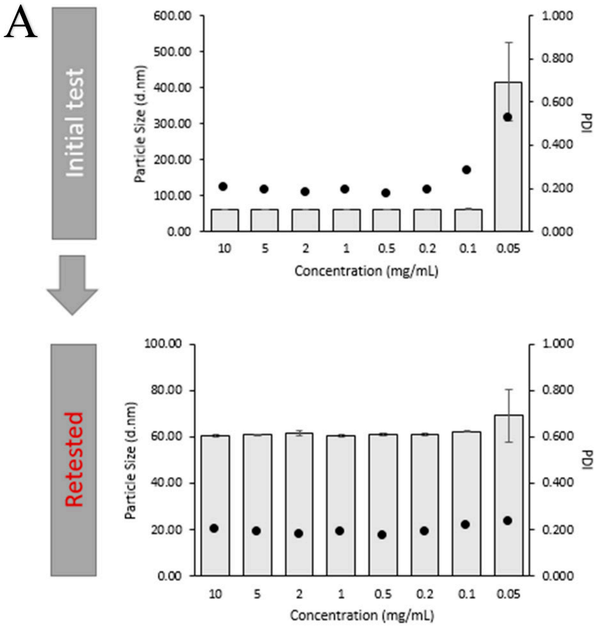

Liposome concentration (mg/mL)	Size (d.nm)	PDI	Derived Count Rate	Attenuation
10	60.61	0.203	298157.23	5
5	60.83	0.191	179646.33	5
2	61.51	0.179	86337.167	6
1	60.58	0.189	42202.7	6
0.5	61.05	0.175	20382.533	7
0.2	61.16	0.192	7605.4333	8
0.1	62.72	0.279	3218.3	8
0.05	415.57	0.522	401.66667	11

Liposome concentration (mg/mL)	Size (d.nm)	PDI	Derived Count Rate	Attenuation
10	60.61	0.203	298157.2	5
5	60.83	0.191	179646.3	5
2	61.51	0.179	86337.17	6
1	60.58	0.189	42202.7	6
0.5	61.05	0.175	20382.53	7
0.2	61.16	0.192	7605.433	8
0.1	62.22	0.219	4217.867	8
0.05	69.23	0.232	1818.1	10

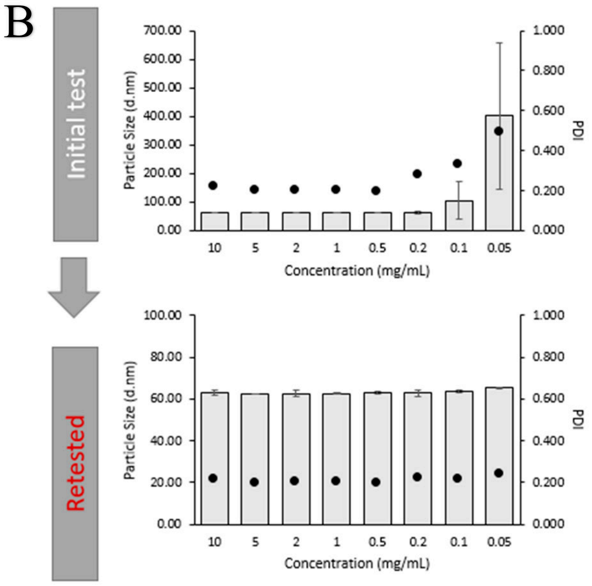

Liposome concentration (mg/mL)	Size (d.nm)	PDI	Derived Count Rate	Attenuation
10	63.11	0.218	290323.7	5
5	62.48	0.198	175091.63	5
2	62.72	0.204	83357.5	6
1	62.63	0.201	43827.1	6
0.5	63.03	0.197	18738.633	7
0.2	63.63	0.279	1608.7667	11
0.1	105.78	0.328	929.26667	8
0.05	401.37	0.493	194.03333	11

Liposome concentration (mg/mL)	Size (d.nm)	PDI	Derived Count Rate	Attenuation
10	63.11	0.218	290323.7	5
5	62.48	0.198	175091.6	5
2	62.72	0.204	83357.5	6
1	62.63	0.201	43827.1	6
0.5	63.03	0.197	18738.63	7
0.2	62.83	0.221	5590.20	8
0.1	63.51	0.215	4371.467	8
0.05	65.28	0.240	2205.4	9

Figure S5: Sensitivity of Malvern Zetasizer. DSPC:Chol:DSPE-PEG2000 liposomes were measured at varying concentrations for size, PDI, Derived count rate and attenuation. A) Shows one batch of liposomes measure at concentrations from 0.05-10 mg/mL, B) shows a second batch of liposomes measured at concentrations from 0.05-10 mg/mL. Demonstrates that the zetasizer is sensitive and accurate at concentrations > 0.2 mg/mL. Each Figure Srepresents n=1.
